# Silver and gold nanoparticles induced differential antimicrobial potential in calli cultures of *Prunella vulgaris*

**DOI:** 10.1186/s13065-022-00816-y

**Published:** 2022-03-25

**Authors:** Nisar Ahmad, Jan Muhammad, Khalil Khan, Wajid Ali, Hina Fazal, Mohammad Ali, Latif-ur Rahman, Hayat Khan, Muhammad Nazir Uddin, Bilal Haider Abbasi, Christophe Hano

**Affiliations:** 1 Centre for Biotechnology and Microbiology, University of Swat, Swat, 19200 Pakistan; 2 Pakistan Council of Scientific and Industrial Research (PCSIR) Laboratories Complex, Peshawar, 25120 Pakistan; 3 Institute of Chemical Sciences, University of Peshawar, Peshawar, 25120 Pakistan; 4 Department of biotechnology, Faculty of Biological Sciences, Quaid-i-Azam University, Islamabad, 45320 Pakistan; 5 Université d’Orléans, Laboratoire de Biologie des Ligneux et des Grandes Cultures (LBLGC), INRA USC1328, 28000 Chartres, France

**Keywords:** *Prunella vulgaris*, Calli, Chemically synthesized silver and gold nanoparticles, Antimicrobial potential

## Abstract

**Background:**

*Prunella vulgaris* is medicinally important plant containing high-valued chemical metabolites like Prunellin which belong to family *Lamiaceae* and it is also known as self-heal. In this research, calli culture were exposed to differential ratios of gold (Au) and silver (Ag) nanoparticles (1:1, 1:2, 1:3, 2:1 and 3:1) along with naphthalene acetic acid (2.0 mg NAA) to investigate its antimicrobial potential. A well diffusion method was used for antimicrobial properties.

**Results:**

Here, two concentrations (1 and 2 mg/6 µl) of all treated calli cultures and wild plants were used against *Escherichia coli, Pseudomonas aeruginosa, Salmonella typhi, Bacillus atrophaeus, Bacillus subtilis, Agrobacterium tumefaciens, Erwinia caratovora* and *Candida albicans.* Dimethyl sulfoxide (DMSO) and antibiotics were used as negative and positive controls. Here, the calli exposed to gold (Au) nanoparticles (NPs) and 2.0 mg naphthalene acetic acid (NAA) displayed the highest activity (25.7 mm) against *Salmonella typhi* than other extracts, which was considered the most susceptible species, while *Agrobacterium tumefaciens* and *Candida albicans* was the most resistance species. A possible mechanism of calli induced nanoparticles was also investigated for cytoplasmic leakage.

**Conclusion:**

From the above data it is concluded that *Prunella vulgaris* is medicinally important plant for the development of anti-microbial drugs using nanotechnology and applicable in various pharmaceutical research.

## Introduction

Nanotechnology is a versatile field and their purpose is increasing rapidly in almost all discipline of sciences. Nanotechnology promotes speedily and estimated that it will revolve toward a trillion-dollar industry at 2021. It has a great influence on industrial revolution [1]. Because of the particular feature of this technology is promoting its approach in different discipline of science. Contrarily, the application of nanotechnology is new in the field of research and it need to progress in plant tissue culture and medicinal plant science. Presently, most of the previous reports with reference to plant growth, seed germination and physiological parameters are anxious regarding to nanoparticles applications [1, 2]. Out of all metal nanoparticles, mainly the gold and silver getting more attention because of their physic-chemical applications [3]. Through silver and gold nanoparticles the growth of microorganisms can be prevented, but this effect is based on the shape and size of nanoparticles [4]. Through gold and silver nanoparticles the genes can be transfer to the target cell in-vitro [5]. The silver and gold nanoparticles are utilized for the identification of protein. The gold nanoparticles are also being useful in the recognition of antigen that is fuse to the antibody [6].

Microbes are evolving to become resistances to particular antibiotics through mutation process [7] and the resistance ability is widespread in microbial population by the process of horizontal gene transfer [8]. Furthermore, as microorganisms become resistant to a particular antibiotic, the subsequent use of alternative antibiotic might select for additional resistance, which give rise to the multi-drug resistance strains [9–11].

The family Lamiaceae contains perennial plant called *Prunella vulgaris.* This is herbaceous plant and used as herbal medicine in many parts of the world. Due to their wound-healing properties, it is also called self-heal or all heal [12]. *Prunella vulgaris* is grown in different areas of Pakistan including Khyber Pokthun Khwa (KPK), Kashmir, Swat and Murree at the height of about 1800 to 3300 m [13]. This medicinal herb is commonly used for the treatment of cold, headache, sore throat and nephritis in many countries including China, Korea and Greece [14]. *Prunella vulgaris* is significant medicinal plant because it having natural phytochemicals. In southeast China, the dried plant is used for drinking as medicine [15].

*Prunella vulgaris* is effective against pain and to minimize the high fever and high blood pressure of the body [16, 17]. Currently, many studies show that this medicinal plant has a wide domain of medicinal properties as anti-cancerous, anti-septic, anti-spasmodic, anti-HSV and anti-rheumatic properties [18, 19]. *Prunella vulgaris* is also effective in case of HIV and herpes viruses and it is functional to treat lymphatic system, and tuberculosis [20]. The best result of *Prunella vulgaris* against bacteria and viruses has been reported. It has been investigated for antimicrobial activities against *E. coli, S. aureus,* and *P. aeruginosa.* This plant has the ability to inhabit many Gram negative bacteria in in-vitro conditions. Prunellin a polysaccharide compound present in this medicinal herb work as repressing against HIV virus [21]. This medicinal herb displayed good effects as anti-mutagenic. The spike of *Prunella vulgaris* taken as a sample and after testing it displayed a good result in opposition to carcinogens and environmental mutagens [22].

Many substances such as flavonoid, coumarins, organic acid, sterols and triterpenes have been extracted from *Prunella.* The aqueous solution of *Prunella vulgaris* containing Prunellin which was then purified and categorized and finally displayed optimal anti-HIV activity [23, 24]. The vulgarisin A, a substance found in this medicinal plant has the ability to inhibit the cancer cell line in human body. Triterpenes substance composed of ursolic acid, oleanolic acid and botulinic acid is also extracted from this plant. Oleanolic acid is effective as anti-inflammatory, anti-fungal as well as helpful in curing lung cancer [25, 26]. *Prunella vulgaris* contain Rosmarinic acid, is a polyphenol act as anti-tumor agent. In *Prunella vulgaris* the bioavailable components characterized which contain tannins (52.25 mg), proteins (441.6), phenolics (55.78 mg), saponins (350 mg) and carbohydrates (375 mg) [27]. But the importance substances of *Prunella vulgaris* are Prunellin and rosmarinic acid [24]. The healing property of this medicinal plant is due to the presence of phenolic acid, while the anti-inflammatory activity is given by the ursolic and oleanolic acid [21]. *Prunella vulgaris* help to block the nerves system inflammation and liver disorder due to the presence of caffeic acid [28].

Many bacteria occur in biofilm, which are the microbial accumulation that gambling on a hard surface and produce extracellular products, including extracellular polymeric substances (EPSs). Normally bacteria make a move reversibly on the surface, but due to the expression of EPSs, the bacteria then attached irreversibly. When the bacteria are attached to the surface, so the formation of the bacterial flagellum is repressed, and the bacteria quickly divide itself and hence formed a mature biofilm. At this point, the bacteria are adhered to each other, developing a block that can show resistance to antibiotics and cause persistent infections. Hence, biofilms remain a major threat to health. Initially, the silver is one of the important metals that are used from the last few decades for its multi-dimensional properties including the antimicrobial potential. But recently, the synthesis of many metal nanoparticles is one of the most important transporting vehicles that cross the cellular membrane and release the required drug for many infectious diseases. So, these metal nanoparticles play a key role in preventing many diseases due to its chemical reactivity, physical strength, optical effects, electrical conductance, and magnetism from the bulk of materials. In this study we also used, the metal nanoparticles in order to inhibit the growth of various biofilm forming microorganisms. As silver and gold is used from ancient times in preventing microbial infection due to selective toxicity to biological systems and is now commonly used in surgical devices, diagnostics, and nanomedicine-based antibacterial agents. Moreover, silver and gold nanoparticles are the most wanted nanomaterials in preventing the microbial infections that form strong biofilms. Unlike biologically synthesized nanoparticles, chemically synthesized nanoparticles may show consistency in dispersity, stability and synthesis protocol. This is the first study, in which an intermediate calli cultures of *P. vulgaris* (exposed to silver and gold nanoparticles) extracts were exploited for antimicrobial potential rather than using nanoparticles directly. The nanoparticles as chemical elicitors will fluctuate the biosynthetic pathways of calli cells and hence it is possible to release higher quantities of antimicrobial agents than wild plant. Therefore, the overall objective of the current study was to investigate the wild plant as well as the extracts of calli cultures exposed to metallic nanoparticles for antimicrobial potential and for possible novel drug designing against resistant bacterial species.

## Materials and methods

### Explant selection and proliferation

The *Prunella vulgaris* healthy and fresh plants were collected from natural habitat of District Swat, Khyber Pokthun Khwa (KPK), Pakistan. These plants were identified and authenticated by Dr. Hina Fazal (Plant Taxonomist) and the specimen with Voucher No. 10500 (PES) has been deposited in the herbarium of Medicinal Botanic Center (MBC), Pakistan Council of Scientific and Industrial Research (PCSIR), Laboratories Complex, Peshawar, Pakistan. Furthermore, all methods were performed (including plant collection) according to institutional and national guidelines which comply with International standards. To develop callus cultures, green and undamaged leaves of *Prunella vulgaris* were excised as optimal explants. The plant leaf was cut into pieces of about 3–4 mm^2^. The explants were sterilized as stated by the procedure of Ahmad et al. [29]. For callus culture, MS media with 2.0 mg/l NAA was used [30]. Then 2.3 g of MS media and 15 g sucrose was added to flask containing distilled water. For solidification of media, 4.5 g agar was added. The flasks containing culture media were sterilized in an autoclave at 121 °C, 15 psi for 20 min. The leaf pieces were incubated onto MS media for 30 days to develop callus. To examine the effect of differential ratios of nanoparticles (NPs) and naphthalene acetic acid (NAA) on callus propagation, fresh callus was taken at the end of 1 month of explants incubation on NAA augmented media. The calli cultures were then shifted to the different fresh media which composed of differential ratios of gold (Au) and silver (Ag) nanoparticles alone which were taken from the experiment of Rahman et al. [31] or combination of NPs with NAA. Rahman et al. [31], reported that the SEM and TEM results revealed that these nanoparticles were mostly uniform with spherical shape and the average size of each proportion ranges from 25 to 35 diameter in nm. Moreover, the different ratios (concentration) were prepared by changing the 0.01 M concentration of silver nitrate and HAuCl_4_ as AuAg 1:1 (0.5 + 0.5), AuAg 1:2 (0.33 + 0.66), AuAg 1:3 (0.25 + 0.75), AuAg 2:1 (0.66 + 0.33) and AuAg 3:1 (0.75 + 0.25) respectively. The stock solution of each nanoparticles was prepared as 1.0 mg/1.0 ml. From the stock solution 30 mg l^−1^ was added to the media along with 2.0 mg/l of NAA according to the protocol of Fazal et al. [30] for callus proliferation. The media and NPs were expressed as treatments in Table 1.

**Table 1:** NPs and NAA applied for callus culture of *prunella vulgaris*

Treatment	NPs + NAA (Auxin)
T1	Full MS with 2.0 NAA
T2	Wild plant
T3	½ MS with 2.0 NAA
T4	AgAu 1:2 + 2.0 NAA
T5	AgAu 1:3 + 2.0 NAA
T6	AgAu 2:1 + 2.0 NAA
T7	AgAu 3:1 + 2.0 NAA
T8	AgAu 1:1 + 2.0 NAA
T9	Au + 2.0 NAA
T10	Ag + 2.0 NAA

To examine the effect of diverse nanoparticles (NPs) and naphthalene acetic acid (NAA) on callus propagation, fresh calli cultures were taken at the end of 1 month of explants incubation on NAA media. The calli cultures were then sub-culture to fresh media which composed of differential ratios of gold (Au) and silver (Ag) nanoparticles alone or in mixture with NAA

### Calli cultures preparation for antimicrobial activities

In this study, to investigate the antimicrobial activities, the calli cultures were taken out from MS media, gently washed the calli with clean distilled water, filter paper was used to eliminate the excess water (Whatman Ltd, England) and then the calli cultures were weighed as a fresh weight (FW). To determine the dry weight (DW), an oven was used to dry the calli (Thermo Scientific; Germany) at 50 °C up to 24 h and the calli were weighed. The dry weight (DW) and fresh weight (FW) of the calli were stated in g/100 ml (Table 2).

**Table 2:** Fresh and dry weight of *Prunella vulgaris* extract

Media used	Callus fresh weight (g)	Callus dry weight (g)
Full MS 2.0 NAA	1.1606 ± 0.07b	0.292 ± 0.012a
Wild plant	0.4972 ± 0.01c	0.0559 ± 0.021b
½ MS 2.0 NAA	1.522 ± 0.087a	0.0321 ± 0.003 c
AgAu 1:2 + 2.0 NAA	0.1770 ± 0.01d	0.0163 ± 0.001e
AgAu 1:3 + 2.0 NAA	0.1275 ± 0.01d	0.0214 ± 0.003d
AgAu 2:1 + 2.0 NAA	0.5323 ± 0.03c	0.0482 ± 0.007b
AgAu 3:1 + 2.0 NAA	0.1905 ± 0.01d	0.0119 ± 0.003e
AgAu 1:1 + 2.0 NAA	0.1311 ± 0.01d	0.0107 ± 0.003e
Au + 2.0 NAA	0.1337 ± 0.01d	0.011 ± 0.001e
Ag + 2.0NAA	0.598 ± 0.04c	0.039 ± 0.0012bc

To know the fresh weight (FW) the inoculated calli were taken and then weighed. For extract preparation, wild plants extract and the fresh calli were dried in an oven and then weighed (DW). All the values are mean ± SE (standard errors). Column labeled with different letters of least significant differences (LSD values) displayed significant variation (α < 0.05)

Fazal et al. [32, 33], protocol was used for the preparation of extract. The callus of *Prunella vulgaris*, were dried in an oven at 50 °C to obtain the dried powder, from which the extract was prepared for anti-microbial activities. Here, 5 g of dried powdered were taken in sterilized flasks. Then 50 ml of ethanol was poured to each flask and incubated for 7 days with periodic shaking and finally filtered. The Whatman filter paper No 1 was utilized for the filtration of final crude extract isolation. The ethanol was only used for the extraction of secondary metabolites from plant-based materials and afterwards completely removed before the final stock solution preparation in another non-toxic solvent. The filtrated extract was collected after I week incubation. The ethanol was removed from the extract by using rotary evaporator at 40 °C. This experiment was 3 times repeated and every time fresh volume of ethanol was used to extract maximum quantities of secondary metabolites. The ethanol was completely removed through rotary evaporator to obtain the crude extract. To concentrate the final extract (ethanol) the rotary evaporator at 40 °C was used, individually for each extract. After evaporation of the ethanol, the extract was kept at 4 °C in a flask and tightly packed it to avoid any reaction or contamination. Finally, the crude extract was dissolved in 0.1% dimethyl sulfoxide (DMSO; 1 mg/μl 6) due its non-toxic nature and it dissolved multiple extracts (prepared in any solvent) as it is considered universal solvent for antimicrobial activities.

### Microorganisms tested for antimicrobial potential

The microorganisms which were used, namely, *Escherichia coli* (*E. coli*; MBC-MIC-003), *Pseudomonas aeruginosa* (*P. aeruginosa*; MBC-MIC-051)*, Salmonella typhi* (*S. typhi*; MBC-MIC-104), *Erwinia caratovora* (*E. caratovora*; MBC-MIC-153) and *Agrobacterium tumefaciens* (*A. tumefaciens*; MBC-MIC-171; Gram negative bacteria) *Bacillus atrophaeus* (*B. atrophaeus* MBC-MIC-203)*, Bacillus subtilis* (*B. subtilis* MBC-MIC-208; Gram positive bacteria)*.* The above bacteria were collected from Pakistan Council of Scientific and Industrial Research (PCSIR) Laboratories Complex, Peshawar, Pakistan. One fungal strain, *Candida albicans* (*C. albicans*; MBC-MIC-304; Gram positive fungus) was used and obtained from the same place (PCSIR, Peshawar, Pakistan). To maintained, microorganisms were placed on solid media at 4 °C till activity (Table 3).

**Table 3:** The microorganisms which were used, namely, *Escherichia coli* (*E. coli*), *Pseudomonas aeruginosa* (*P. aeruginosa*)*, Salmonella typhi* (*S. typhi*), *Erwinia caratovora* (*E. caratovora*) and *Agrobacterium tumefaciens* (*A. tumefaciens*; Gram negative bacteria) *Bacillus atrophaeus* (*B. atrophaeus*)*, Bacillus subtilis* (*B. subtilis*)*,* (Gram positive bacteria)*.* One fungal strain, *Candida albicans* (Gram positive fungus)

Microorganisms	Type	Authentications No.	Source
*Escherichia coli*	Gram negative	MBC-MIC-003	PCSIR Lab (Peshawar)
*Pseudomonas aeruginosa*	Gram negative	MBC-MIC-051	PCSIR Lab (Peshawar)
*Salmonella typhi*	Gram negative	MBC-MIC-104	PCSIR Lab (Peshawar)
*Bacillus atrophaeus*	Gram positive	MBC-MIC-203	PCSIR Lab (Peshawar)
*Bacillus subtilis*	Gram positive	MBC-MIC-208	PCSIR Lab (Peshawar)
*Agrobacterium tumefaciens*	Gram negative	MBC-MIC-171	PCSIR Lab (Peshawar)
*Erwinia caratovora*	Gram negative	MBC-MIC-153	PCSIR Lab (Peshawar)
*Candida albicans*	Fungi	MBC-MIC-304	PCDIR Lab (Peshawar)

The above microorganisms were collected from Pakistan Council of Scientific and Industrial Research (PCSIR) Laboratories Complex, Peshawar, Pakistan

### Determination of anti-microbial potential of NPs induced calli cultures

The anti-microbial ability of callus of *Prunella vulgaris* extract was determined through Well-diffuse method [32–34]. In this method the microbe’s suspension were added to nutrient agar media and level off the microbial suspension through sterile swab moistened. In each agar media plate 4 wells of about 4 mm in diameter were boarded through a sterilized cork-borer. Then 24 μl of 0.1% dimethyl sulfoxide (DMSO) was added to one well of each inoculated media plate as negative control. In second well 24 μl of 2.0 mg/6 μl antibiotics (the antibiotics which was effective against the microbe), were poured to each media plates as positive control. For positive control, broad spectrum antibiotics including Azithromycin, Ciprofloxacin, Streptomycin and Clotrimazole were used. The Ciprofloxacin was used against *E. coli*, *B. atrophaeus* and *B. subtilis*. The Azithromycin was used against *P. aeroginosa* and *S. typhi*. The Clotrimazole was used against *C. albicans* and the Streptomycin was generally applied against *A. tumefaciens* and *E. caratovora* respectively. In the third well of all agar media plates were filled with 1 mg of each solvent extract and then to the fourth well 2 mg of each solvent extract to all agar media plates were added. After this procedure the inoculated agar media plates were kept at 32–37 °C for 24 h. The 0.1% DMSO did not show any activity while, antibiotics and solvent extract inhibit the growth of microorganisms, which were present in media. Zones were formed around those wells where the growth of microorganisms was inhibited. These zones were measured in millimeter.

### Statistical analysis

The mean zone of inhibition was obtained from triplicated data using Statistix software (V 8.1; USA). One-way analysis of variance (ANOVA) was used for mean determination. The standard errors (SE) of the mean did not exceed 5%. The least significant differences (LSD) values was also obtained using Statistix software (V 8.1; USA).

## Results

In the current research, the callus cultures of medicinally important *Prunella vulgaris* were grown in PCSIR Peshawar. The wild plants were collected from wild habitat and washed with distilled water to remove the dust particles. In PCSIR Labs Complex, Peshawar-Pakistan, the explants (leaves) were sterilized with 70% ethanol and 0.5–2% NaOCl. The callus was cultured on Murashige and Skoog media containing 2.0 mg/l of naphthalene acetic acid (NAA). To study the out-come of nanoparticles (gold and silver NPs) and naphthalene acetic acid (NAA), the fresh callus was then sub-culture to agar media containing gold and silver NPs and NAA. In this study, 9 different treatments were tested with NAA either NPs alone or in differential ratios to determine the efficiency of callus proliferation. It was obtained that the calli responded to all treatments containing NPs and NAA. To know the fresh weight (FW), the NPs treated calli cultures were taken and then weighed. For extract preparation, wild plants extract and the fresh NPs treated calli were dried in an oven and then weighed (DW). The fresh and dry weight was shown in Table 2. The extract was prepared for antimicrobial activities and the samples were used against Gram negative bacteria (*E. coli, P. aeruginosa, S. typhi, A. tumefaciens* and *E. caratovora*, Gram positive bacteria (*B. atrophaeus and B. subtilis* and a fungus (*C. albicans*). Dimethyl sulfoxide (0.1%; DMSO) and antibiotics were also applied against the microbes for negative and positive controls.

### Nanoparticles induced differential antimicrobial potential in Calli cultures of *Prunella*

In this study, the ethanolic extract was investigated for antimicrobial potential. However, during extract preparation for antimicrobial activities, the ethanol was completely removed from the extract and the final extract was dissolved in 0.1% DMSO which did not exhibit any antimicrobial activity. Here, the antimicrobial activities of calli cultures of *P. vulgaris* was investigated. The main aim of the study was to investigate the differential antimicrobial potential of calli cultures using different treatments. For more clarity and to obtain the actual antimicrobial results, the wild plants, plant growth regulator (NAA)-induced calli cultures and the calli cultures-induced by different ratios of NPs along with NAA were investigated independently. The wild plants, full MS media + 2.0 mg/l NAA induced calli, half MS media + 2.0 mg/l NAA induced calli, calli cultures induced by NAA + NPs (1:1, 1:2, 1:3, 3:1, and 2:1) were investigated for the antimicrobial activities. The NPs alone was not used for callus induction and subsequent antimicrobial activities, because without plant growth regulators, the NPs alone cannot induce or proliferate the calli cultures. These different treatments (Table 1) were designed to investigate that whether the activities are due to NAA alone or due to the NPs alone, or due to the combination of NPs + NAA and in comparison, wild plants were also investigated to get the final results (Table 4).

**Table 4:** Antimicrobial potential of calli cultures of *Prunella vulgaris* induced by NAA and nanoparticles

Media used	Sample weight	Extract weight	mg/ul	*E.c*	*P.a*	*S. t*	*B.a*	*B.s*	*A.t*	*E.c*	*C.a*
Full MS 2.0 NAA	1.1606	0.292	1 mg	–	–	10 ± 0.2d	–	–	–	–	–
			2 mg	–	10.4 ± 0.1c	10.3 ± 0.1d	–	15.3 ± 0.2c	–	10.6 ± 0.1d	–
Wild plants	0.4972	0.0559	1 mg	10.5 ± 0.3b	20 ± 0.2b	10.6 ± 0.3d	–	10.5 ± 0.3d	–	–	10.9 ± 0.3b
			2 mg	10.5 ± 0.2b	20.4 ± 0.2b	15.3 ± 0.1c	10.2 ± 0.1d	15 ± 0.1c	20.4 ± 0.3b	10 ± 0.2d	–
½ MS 2.0 NAA	1.522	0.0321	1 mg	–	10.7 ± 0.3c	10.4 ± 0.2d	10 ± 0.1d	9.9 ± 0.3d	20.2 ± 0.3b	20.5 ± 0.3b	–
			2 mg	–	–	10.5 ± 0.3d	9.9 ± 0.2d	15.4 ± 0.2c	–	–	–
AgAu 1:22.0 NAA	0.1770	0.0163	1 mg	–	–	10.2 ± 0.1d	–	–	–	–	–
			2 mg	10.3 ± 0.2b	–	10.1 ± 0.1d	–	10.5 ± 0.1d	–	–	–
AgAu 1:32.0 NAA	0.1275	0.0214	1 mg	9.9 ± 0.2b	–	–	–	–	–	10.3 ± 0.1d	–
			2 mg	10.2 ± 0.3b	–	–	15.3 ± 0.3c	10.3 ± 0.2d	–	9.9 ± 0.2d	–
AgAu 2:12.0 NAA	0.5323	0.0482	1 mg	–	10.8 ± 0.3c	10.7 ± 0.2d	10.1 ± 0.1c	15.1 ± 0.3c	–	10.5 ± 0.2d	–
			2 mg	–	–	15.3 ± 0.3c	15.4 ± 0.1c	20.5 ± 0.4b	–	15.3 ± 0.3c	–
AgAu 3:12.0 NAA	0.1905	0.0119	1 mg	–	–	10.3 ± 0.1d	10.2 ± 0.2d	–	–	15.1 ± 0.3c	–
			2 mg	–	–	10.4 ± 0.1d	15.3 ± 0.2c	–	–	–	–
AgAu 1:12.0 NAA	0.1311	0.0107	1 mg	–		–	–	–	–	–	–
			2 mg	5.3 ± 0.1c		5.0 ± 0.1e	–	6.1 ± 0.2e	–	7.1 ± 0.2d	–
Au2.0 NAA	0.1337	0.011	1 mg	9.9 ± 0.1b	–	10.9 ± 0.2d	–	–	–	–	–
			2 mg	10.3 ± 0.2b	–	25.7 ± 0.3b	–	–	–	–	–
Ag2.0 NAA	0.598	0.039	1 mg	10 ± 0.1b	–	10.3 ± 0.1d	20 ± 0.3b	10.3 ± 0.1d	–	–	10.8 ± 0.2b
			2 mg	10.3 ± 0.2b	–	10.5 ± 0.1d	25 ± 0.3ab	10.5 ± 0.1d	–	–	–
DMSO	–	–	–	–	–	–	–	–	–	–	–
Antibiotics (Az, Cip, Clot and Strep			2 mg	22.1 ± 0.4a	28 ± 0.3a	34 ± 0.5a	27 ± 0.4a	28.7 ± 0.3a	30 ± 0.4a	28 ± 0.5a	30.1 ± 0.4a

(***E.c***) *E. coli*, (***P.a***) *P. aeruginosa*, (***S. t***) *S. typhi*, (***B.a***) *B. atrophaeus*, (***B.s***) *B. subtilis*, (***A.t***) *A. tumefaciens*, (***E.c***) *E. caratovora* and (***C.a***) *C. albicans.* All the values are mean ± SE (standard errors). Column labeled with different letters of least significant differences (LSD values) displayed significant variation (α < 0.05)

In this study two different concentrations (1 mg/6 ul and 2 mg/6 ul) of wild plant extracts, NPs-treated calli cultures and callus grown on half and full MS media augmented with 2 mg/l NAA alone was compared for antimicrobial potential against human and plant pathogens. The 1 mg/6 ul extract of full MS media with 2 mg/l NAA was only effective against *S. typhi* and displayed 10 mm zone of inhibition. The 2 mg/6 ul extract was more effective against *B. subtilis* and formed 15.25 mm zone of inhibition while, against *E. caratovora,* it displayed 10.65 mm zone respectively. The 2 mg/6 ul extract of wild plant have shown maximum activities against *P. aeruginosa* and *A. tumefaciens* (20.4 mm zone of inhibitions). Furthermore, the 1 and 2 mg/6 ul extracts obtained from calli induced by Half MS media with 2.0 mg/l NAA were useful and inhibited the growth of *S. typhi* (10.4 mm and 10.55 mm), *B. atrophaeus* (10 mm and 9.95 mm) and *B. subtilis* (9.95 mm and 15.35 mm). Interestingly, the 1 mg/6 ul extract of Half MS media was more active against plant pathogens including *A. tumefaciens* (20.15 mm) and *E. caratovora* (20.5 mm) as shown in Table 4.

Both the extract concentrations of calli obtained from NPs (AgAu 2:1) and NAA (2.0 mg/l) augmented media did not show antimicrobial activities against *E. coli* and *A. tumefaciens,* as well as fungus (*C. albicans*)*.* The same extracts exhibited optimal activities of 10.7 mm and 15.25 mm against *S. typhi*, while the extracts displayed 10.1 mm and 15.4 mm zones of inhibition against *B.* atrophaeus. However, the extracts displayed 15.1 mm and 20.55 mm, and 10.5 mm and 15.25 mm zones of inhibition against *B. subtilis* and *E. caratovora* respectively. The antimicrobial activity of the calli extract grown on higher ratio of NPs (AgAu 3:1) and NAA (2.0 mg/l) were applied against pathogenic microorganisms. Both extracts showed antimicrobial activities and inhibited the growth of *S. typhi* (10.25 mm and 10.35 mm) and *B. atrophaeus* (10.2 mm and 15.3 mm), while *E. caratovora* (15.1 mm) was only inhibit by the 1 mg/6 ul (Table 4). The silver nanoparticles alone in combination with NAA was more effective against *B. atrophaeus* and displayed 20 mm and 25 mm zones of inhibition respectively. Further, 10.25 mm and 10.5 mm inhibition zones were observed against *B. subtilis*. Here, 1 mg/6 ul extract showed 10.85 mm activity against the fungal strain (*C. albicans*). The calli extract which were taken from gold NPs and NAA (1 and 2 mg/6 ul) displayed inhibitory potential against *E. coli* (9.9 mm and 10.25 mm) and *S. typhi* (10.9 mm and 25.7 mm). The 2 mg/6 ul extract exhibited the highest antimicrobial potential of 25.7 mm against the pathogenic *S. typhi* in this research. The antimicrobial activities of both extracts (1 and 2 mg/6 ul) were less effective against the bacterial strains including *P. aeruginosa, B. atrophaeus, B. subtilis, A. tumefaciens* and *E. caratovora* as well as to the fungal strain (*C. albicans*; Table 4). The calli cultures exposed to AgAu NPs (1:2) and NAA displayed minimum activities against pathogenic bacteria. This extract did not show any potentiation to the other microbes used in this study. The activity of 2 mg/6 ul extract of calli was also less effective, but maximum than the 1 mg/6 ul. The highest inhibition rate of this extract was observed against *E. coli* (10.25 mm), *S. typhi* (10.1 mm) and *B. subtilis* (10.5 mm) as shown in Table 4. The antimicrobial activity of 1 mg/6 ul AgAu NPs (1:3) and NAA extracted calli were investigated against multiple microbes and displayed activities against *E. coli* (9.9 mm) and *E. caratovora* (10.25 mm), while no such results was found against the remaining strains of bacteria and fungus. The activity of 2 mg/6 ul was found to be effective than 1 mg/ul. Its highest effect was recorded against *B. atrophaeus* (15.25 mm), *B. subtilis* (10.25 mm), *E. coli* (10.2 mm) and *E. caratovora* (9.9 mm). The extract of this cultures did not show any effect against *P. aeruginosa, S. typhi,* and *A. tumefaciens*, and to the fungal strain (*C. albicans*)*.* The zones of inhibition of positive control (Azithromycin, Ciprofloxacin, Streptomycin and Clotrimazole) was also measured. Here, the Ciprofloxacin (2.0 mg/6 μl) displayed 22.1 mm zone of inhibition against *E. coli*. The same antibiotic exhibited 27 mm zone of inhibition against *B. atrophaeus* and 28 mm zones of inhibition against *B. subtilis*. The Azithromycin displayed 28 mm zone of inhibition against *P. aeroginosa*, and 34 mm against *S. typhi* (Table 4). The Clotrimazole also inhibited the growth of *C. albicans* (30.1 mm zone of inhibition). However, the Streptomycin was generally applied against *A. tumefaciens* and *E. caratovora* which displayed 30 mm and 28 mm zones of inhibitions.

Moreover, the calli cultures treated with nanoparticles reduced the fresh and dry biomass but contrarily, according to the objective of the study, the nanoparticles treated calli cultures displayed maximum antimicrobial potential than wild plants as well as untreated calli cultures. Hence nanoparticles play a key role in the enhanced production of phytochemicals that is responsible for antimicrobial potential.

## Discussion

**Fig. 1:**
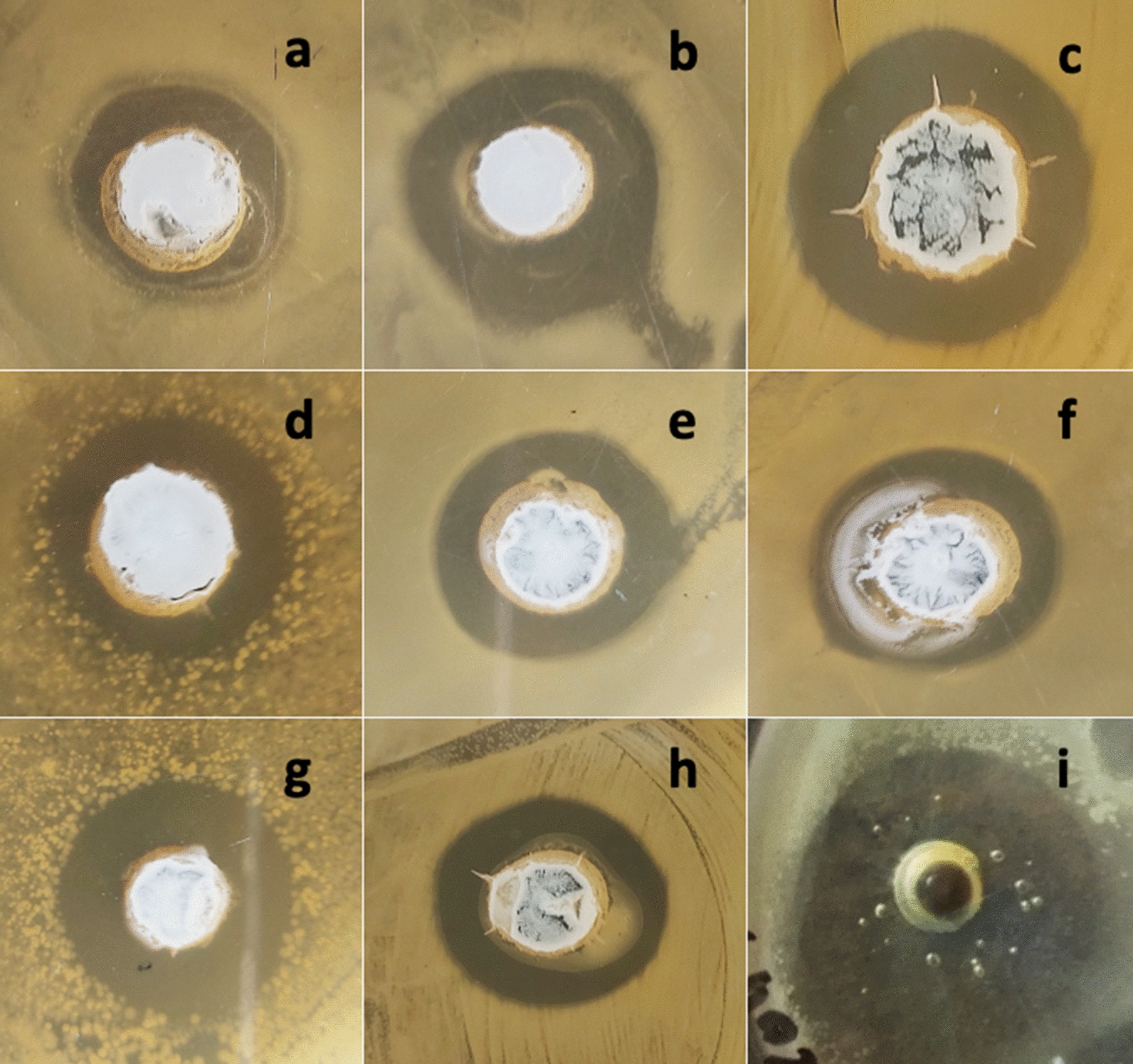
Pictorial presentation of antimicrobial potential of differential ratios of nanoparticles, wild plant, untreated calli cultures and calli cultures of *Prunella vulgaris* (**a**) effect of AgNPs with NAA against *E. coli* (**b**) wild plant against *Pseudomonas aeroginosa* (**c**) AuNPs with NAA against *Salmonella typhi* (**d**) AgNPs with NAA against *Bacillus atrophaeus* (**e**) AgAuNPs (2:1) with NAA against *Bacillus subtilis* (**f**) wild plant against *Agrobacterium tumefaciens* (**g**) untreated calli cultures against *Erwinia caratovora* (**h**) AgNPs with NAA against *Candida albicans* (**i**) control

Microorganisms are the active sources of causing different types of disease in human, plants and animals. Earlier effective antibiotics were used against microorganisms but those antibiotics are not effective in the current era, because antibiotics resistance mechanism were developed by these pathogenic microorganisms. The resistance mechanism in microbes is developing by mutation or genetic recombination [35]. In this study we focus on medicinal plant (*Prunella vulgaris*) extract alone or the calli grown on MS media augmented with different ratios of NPs and NAA which have displayed strong antimicrobial activities (Fig. 1). The medicinal plant extract is less dangerous to the health of humans and animals [36]. The explant derived from leaf of *Prunella vulgaris* and its subsequent proliferation to callus was exploited as antimicrobial agent. The calli were than exposed to different concentration of gold (Au) and silver (Ag) nanoparticles alone and with the combination of 2.0 mg/l NAA (auxin). Yet it is not understandable that how these gold and silver NPs alone or along with NAA control callus growth in medicinal plant including *P. vulgaris* [37]. The present research aimed to determine the antimicrobial ability of various extract obtained from *P. vulgaris* against seven pathogenic bacterial strains and one fungal strain. The antimicrobial activities of *P. vulgaris* plant and calli extracts obtained from different media used in this research was determined by measuring the inhibition zones of two concentrations (1 and 2 mg/6 μl).

**Fig. 2:**
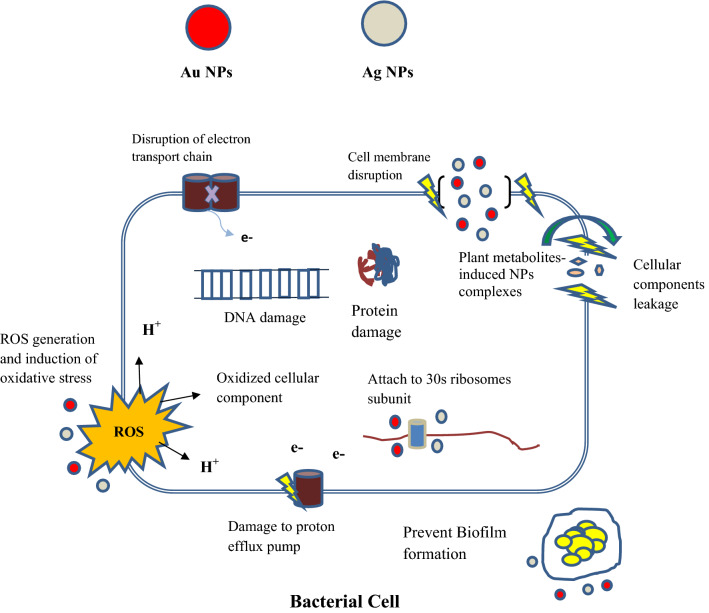
Different mechanisms of action of NPs in bacterial cell. The gold and silver NPs interact with MDR microbes and activate oxidative stress mechanisms. It also inhibits enzymes and proteins as well as epigenetic changes in DNA.

In the current study, the most significant activity of the extract obtained from wild plant which was effective against all of the bacterial species and fungi; however, Rasool et al. [38], also proposed that the wild plant is more effective against pathogenic microbe, while the combination of gold (Au) and 2.0 mg NAA was only effective against *E. coli* and *S. typhi*, as Krutyakov et al. [39] proposed that silver NPs have better antimicrobial activity than gold. The AgAu (1:3) with 2.0 mg NAA combination was less effective against *S. typhi,* while the other extract showed activities against *S. typhi*, as Yallappa et al. [40] reported that Ag, Au and Ag-Au NPs showed antimicrobial activities against *S. typhi.* The growth of *C. albicans* was only inhibited by 1 mg/6 μl extract of wild plant, where 10.9 mm of zone was appeared and the 1 mg/6 μl extract of Ag + 2.0 mg/l NAA displayed 10.85 mm zone of inhibition, while *C. albicans* show resistance to the other extracts, as Yallappa et al*.* [40] suggest that Ag NPs exhibited optimum antimicrobial activities than Au NPs on fungal strains. The combination of Au + 2.0 mg/l NAA effect the growth of *S. typhi* and exhibited the highest inhibition zone of 25.7 mm. Tabrizi et al. [41] reported that the antimicrobial activities of synergistic combination of Ag-Au are more effective than Ag and Au alone. From the above statement it is observed that the microbial strains *S. typhi* and *B. subtilis* are more susceptible to the *Prunella vulgaris* extract, while Yallappa et al*.* [40] and Bankura et al. [42] reported that *S. typhi* and *B. subtilis* are more susceptible to Ag-Au NPs. The bacterial strain *A. tumefaciens* and fungal strain (*C. albicans*) are more resistance to the different extract of calli grown on multiple media containing combinations of NPs and NAA*.* Nanoparticles utilized their antimicrobial activities through several mechanisms, including: (1) direct interaction with microbial cell wall; (2) prevention of Biofilm generation; (3) initiation of reactive oxygen species (ROS); and (4) interaction with DNA or proteins [43]. The gold and silver NPs interact with MDR microbes and activate oxidative stress mechanisms. It also inhibits enzymes and proteins as well as epigenetic changes in DNA [44]. The Ag NPs interact with cell membrane and increase their permeability. Ag NPs activate ATP generation and DNA replication in microbes [45]. The Au NPs interact with bacterial cell membrane and lead to cell lysis [46]. To protect from antibiotics the bacteria form Biofilm, in which the bacterial cells are attaching to each other on the surface within extracellular polymeric penetration (EPS) produce by the bacterial cell. EPS help the bacterial cell to become resistances to multiple antibiotics [47]. Most of the studies suggest that NPs prevent Biofilm formation by penetrating the EPS matrix and cause death of the microorganisms [48] as shown in Fig. 2. Therefore, the nanoparticle induced calli culture of *P. vulgaris* release metabolites of interest such as antimicrobial component which enhanced the activity of these metabolites along with nanoparticles as possible complexes and attached to the bacterial cell. Due to the nano size of nanoparticle complexes, it penetrates into the bacterial cell, disturb the gene expression, efflux pump and cause oxidative ROS to burst the cell membrane and to produce pores in bacterial cell/or cytoplasmic leakage which ultimately killed the pathogenic microbial cell.

## Conclusion

Medicinal plant *Prunella vulgaris* is broadly distributed in many countries of the World. This medicinal plant has ironic properties with lower-caste and well performing than synthetic drugs. However, no one studied the effect of elicitors (Ag/Au NPs) on intermediate plant cells (calli cultures) that is not present naturally but induced by plant growth regulators in-vitro. The callus is undifferentiated mass of cells and every cell in the callus have the ability of totipotency. These totipotent cells produce metabolites of interest like that of mother plant. The addition of elicitors to the culture media containing plant growth regulators fluctuate the biosynthetic pathways and sometimes release higher quantities of secondary metabolites than mother plant. Here, the calli induced by different ratios of nanoparticles displayed higher activities than wild plants and callus grown on media containing plant growth regulators but no nanoparticles. Such studies elaborate the traditional knowledge of the medicinal plant and here this study proved that either whole plant or plant cells have the ability to control the spreading of resistant strains. In this study we notice that the extract of *Prunella vulgaris* alone or calli cultures having a strong antimicrobial activity against different bacteria and fungi. It shows that the compound present in this medicinal plant inhibits the microbial growth. So, the *Prunella vulgaris* can be very useful in making new anti-microbial drugs, and anti-cancerous drugs. Due to its anti-microbial, anti-cancerous and healing properties it gets more attention in different field of biological research.

## Data Availability

Data related to this manuscript will be available on request to the corresponding authors.

## Abbreviations

FW: Fresh weight
DW: Dry Weight
Au: Gold
Ag: Silver
NPs: Nanoparticles
NAA: Naphthalene acetic acid
mm: Millimeter
PCSIR: Pakistan Council of Scientific and Industrial Research
MDR: Multi drug resistance
LSD: Least significant differences
MS-media: Murashige and Skoog media
DMSO: Dimethyl sulfoxide
*A. tumefaciens*: *Agrobacterium tumefaciens*
*B. atrophaeus*: *Bacillus atrophaeus*
*B. subtilis*: *Bacillus subtilis*
*C. albicans*: *Candida albicans*
E. coli: *Escherichia coli*
*E. caratovora*: *Erwinia caratovora*
*P. aeruginosa*: *Pseudomonas aeroginosa*
*S.** aureus*: *Staphylococcus aureus*
